# Supplementary figures and images for: Seroprevalence of antibodies against Chlamydia trachomatis and enteropathogens and distance to the nearest water source among young children in the Amhara Region of Ethiopia
Source: PLoS Negl Trop Dis. 2020 Sep 2;14(9):e0008647. doi: 10.1371/journal.pntd.0008647 (PMC7491729; doi:10.1371/journal.pntd.0008647)

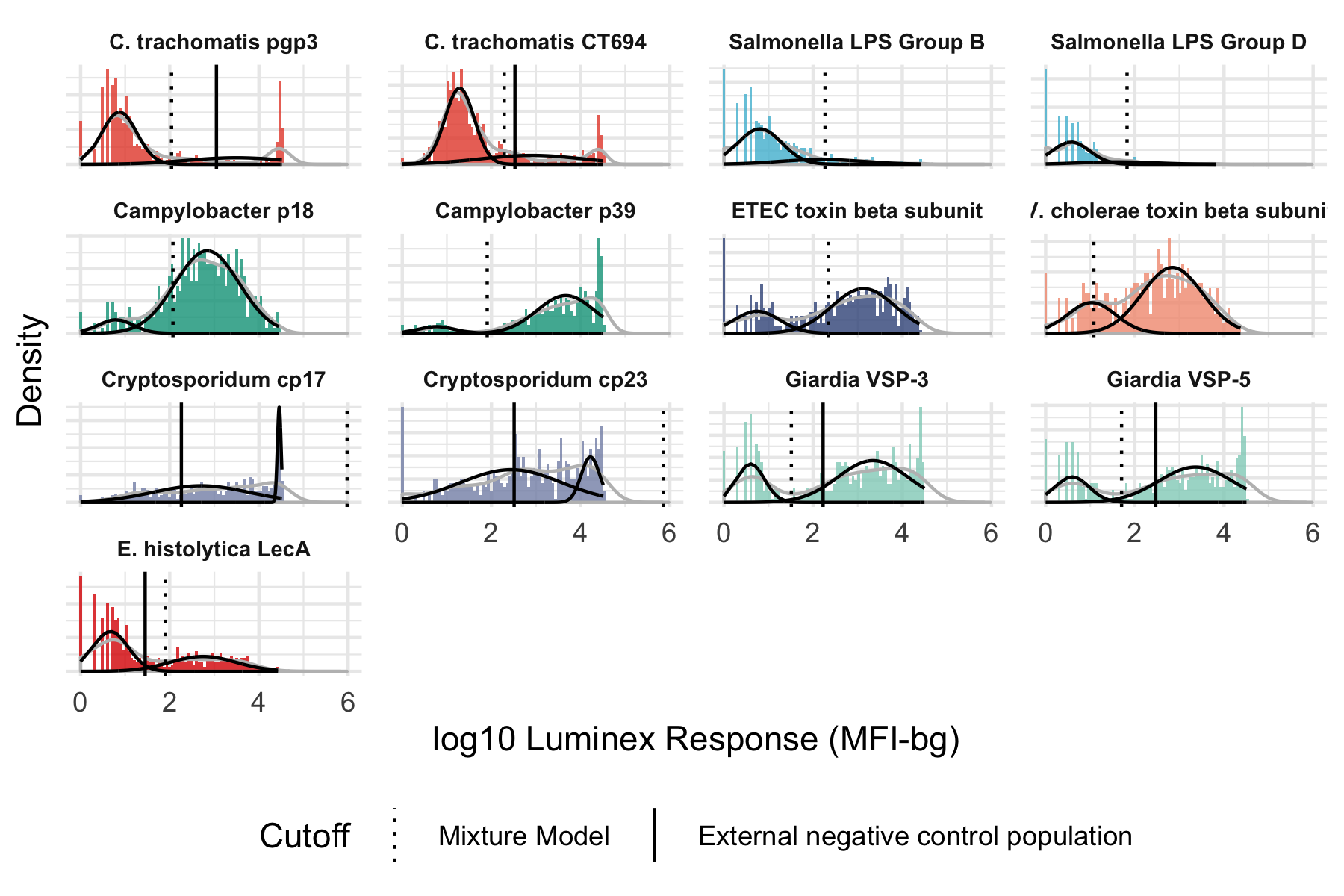

Supplement: S1 Fig — IgG antibody response measured in multiplex using median fluorescence units minus background (MFI-bg) on the Luminex platform. Population restricted to children <24 months to derive cutoffs (n = 317). Vertical lines mark seropositivity cutoffs based on external negative controls (solid) and finite Gaussian mixture models (dash). For Chlamydia trachomatis pgp3 & CT694 cutoffs were derived using receiver operating characteristic (ROC) curves, for Cryptosporidium parvum Cp17 & Cp23 cutoffs were derived using a standard curve and for Giardia intestinalis VSP-3 & VSP-5 and Entamoeba histolytica LecA cutoffs were derived using the mean plus 3 standard deviations above a negative control panel. (TIFF) [file pntd.0008647.s001.tiff]

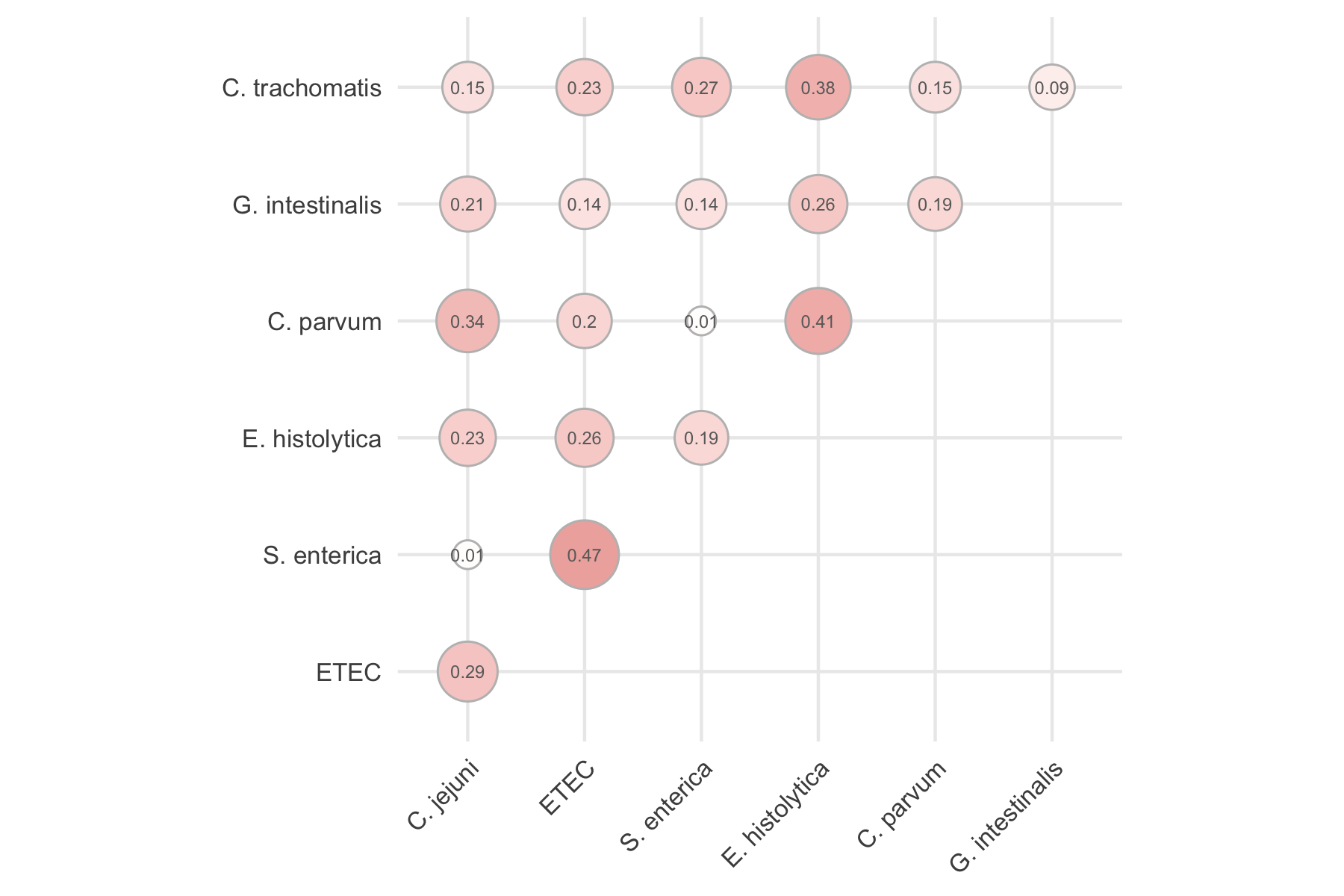

Supplement: S2 Fig — Correlation between the mean community seroprevalence depicted with circles, greater circle area represents higher correlation. For pathogens with more than one antigen, positivity to either antigen was considered positive. IgG response measured in multiplex using median fluorescence units minus background (MFI-bg) on the Luminex platform on 2267 blood samples from 2267 children aged 0 to 9 years. (TIFF) [file pntd.0008647.s002.tiff]
