# Supplementary material for: Seroprevalence of antibodies against Chlamydia trachomatis and enteropathogens and distance to the nearest water source among young children in the Amhara Region of Ethiopia
Source: PLoS Negl Trop Dis. 2020 Sep 2;14(9):e0008647. doi: 10.1371/journal.pntd.0008647 (PMC7491729; doi:10.1371/journal.pntd.0008647)
Supplement: S1 Table — (DOCX) [file pntd.0008647.s003.docx]

**Supplemental Table 1: Quantitative antibody levels by distance quartile and differences comparing Quartile 4 to Quartile 1**

|  | **Quantitative antibody levels according to distance to the nearest water source (quartiles)** | | | |  |  |
| --- | --- | --- | --- | --- | --- | --- |
|  | **Q1** | **Q2** | **Q3** | **Q4** |  |  |
| **Antigen** | **N=566**  **(Age <3, n=134)** | **N=568**  **(Age <3, n=138)** | **N=564**  **(Age <3, n=123)** | **N=565**  **(Age <3, n=125)** | **Q4 vs Q1 ΔMFI-bg (95% CI)** | **P-value** |
| C trachomatis pgp3 | 2.31 | 2.30 | 2.33 | 2.46 | 0.15 ( -0.22, 0.52) | 0.437 |
| C. trachomatis CT694 | 2.17 | 2.11 | 2.15 | 2.23 | 0.06 ( -0.23, 0.35) | 0.670 |
| Salmonella LPS Group B | 1.48 | 1.59 | 1.67 | 1.67 | 0.19 ( -0.02, 0.39) | 0.076 |
| Salmonella LPS Group D | 0.98 | 1.14 | 1.24 | 1.29 | 0.30 (0.12, 0.48) | 0.001 |
| Campylobacter p18** | 2.40 | 2.40 | 2.40 | 2.38 | -0.00 (-0.11, 0.1) | 0.937 |
| Campylobacter p39** | 3.03 | 3.07 | 3.01 | 2.99 | -0.02 ( -0.13, 0.08) | 0.650 |
| ETEC toxin beta subunit** | 2.75 | 2.85 | 2.78 | 2.83 | 0.08 ( -0.08, 0.25) | 0.323 |
| V. cholerae toxin beta subunit** | 2.61 | 2.70 | 2.65 | 2.71 | 0.09 ( -0.06, 0.25) | 0.237 |
| Cryptosporidum cp17** | 3.49 | 3.57 | 3.62 | 3.57 | 0.07 ( -0.09, 0.23) | 0.406 |
| Cryptosporidum cp23** | 3.27 | 3.32 | 3.32 | 3.30 | 0.02 ( -0.12, 0.16) | 0.728 |
| Giardia VSP-3** | 2.30 | 2.34 | 2.32 | 2.39 | 0.11 ( -0.04, 0.26) | 0.149 |
| Giardia VSP-5** | 2.36 | 2.39 | 2.36 | 2.43 | 0.09 ( -0.07, 0.25) | 0.268 |
| E. histolytica LecA** | 2.03 | 2.07 | 1.94 | 1.99 | -0.05 ( -0.24, 0.15) | 0.642 |
| All prevalence difference estimates are adjusted for age and account for variation in the standard error due to clustering by community. ** Age restricted to 0-3 years Quartile 1 (Q1): 11.4m - 267m; Quartile 2 (Q2): 268m - 472m; Quartile 3 (Q3): 473m - 720m; Quartile 4 (Q4): 721 - 2906m | | | | | | |
