# Supplementary material for: Seroprevalence of antibodies against Chlamydia trachomatis and enteropathogens and distance to the nearest water source among young children in the Amhara Region of Ethiopia
Source: PLoS Negl Trop Dis. 2020 Sep 2;14(9):e0008647. doi: 10.1371/journal.pntd.0008647 (PMC7491729; doi:10.1371/journal.pntd.0008647)
Supplement: Attachment — Submitted filename: SwiftSero_PlosNTD_R1_Response to Reviewers_07.21.20.pdf [file pntd.0008647.s004.pdf]

Department of Medicine
Division of Infectious Diseases and Geographic Medicine

Jeremiah M. Ngondi, MB.ChB, MPhil, MFPH, Ph.D
Associate Editor
PLOS Neglected Tropical Diseases

Elsio Wunder Jr
Deputy Editor
PLOS Neglected Tropical Diseases

July 21, 2020

Dear Drs. Ngondi and Wunder:

Thank you considering our manuscript "**Seroprevalence of antibodies against Chlamydia trachomatis and enteropathogens and distance to the nearest water source among young children in the Amhara Region of Ethiopia**" for publication in *PLOS Neglected Tropical Diseases.* We found the reviewer comments valuable and have addressed them below.

All authors have seen and approved the revision. The manuscript has not been previously published nor is it being considered for publication elsewhere. We have no conflicts of interest.

A pre-print of the manuscript was submitted to medRvix on 04.10.20 (MS ID#: MEDRXIV/2020/060996)

Corresponding author contact information:

Kristen Aiemjoy, PhD MSc
1 808 227 7633
kaiemjoy@stanford.edu
Lane Building, Suite 134, Room 141
300 Pasteur Drive
Stanford, CA 94305-5107

Sincerely,

Kristen Aiemjoy

Kristen Aiemjoy, PhD MSc

Here is the response to the request for modifications sent on 07/21/20:

-Please specify whether every participant under the age of 18 had consent provided by a parent or legal guardian.
Yes, all participants under the age of 18 had consent provided by a parent or guardian

-Please specify whether the IRB specifically approved the use of oral consent for this study.
Yes, The IRB specifically approved the use of oral consent for this study.

We added the following to clarify the ethics statement: "Oral consent was approved by all the institutional review boards and was obtained from each participant or their guardian for participants younger than 18 years."

Revision 1 Response:

Reviewer #1: Methods: It would be helpful to know a bit more about the study location, not just that it's rural. Are pastoral activities common? Climate? Extreme water scarcity is mentioned in the last sentence of the discussion, but more could be said in the methods about the context.

Response: We added the following sentences to the methods: "Most of the rainfall in the Wag Hemra zone occurs in June, July and August, however there is significant seasonal and interannual variability, predisposing the region to drought [12]. The topography is mountainous with steep gorges and valleys."

Is "community" the cluster unit? I assume so, but please clarify.

Response: We clarified that communities are the cluster unit in the following sentence: "We conducted a cross-sectional study evaluating antibody responses in children at the baseline visit of a cluster-randomized trial of a water, sanitation and hygiene (WASH) intervention in 40 communities (the cluster unit) in the Amhara region of Ethiopia."

Study population, second paragraph, first sentence:
"in December 2015, approximately one month before the baseline examination visit" please clarify, one month before the baseline examination visits began, as it appears the baseline took 4 months to conduct. This time gap is important to note, as the authors later highlight that the list of sources according to may not reflect sources actually used in the month(s) prior to the dried blood spot collection.

Response: We clarified the sentence as follows: "Study staff performed a door-to-door census in December 2015, approximately one month before the baseline examination visit began."

Study population, second paragraph, last sentence: Though not the focus of this study, please clarify how the primary outcome for the overall trial was determined? And please make more clear in this sentence that the sample size was therefore not calculated for the intention of performing the analyses presented in this manuscript (correct?).

Response: We added additional detail to the following sentence: "The sample size was calculated for the primary outcome of the trial (molecular detection of ocular C. trachomatis infection)."

Measurements, dried blood spots paragraph – please clarify length of time samples stored at -20C in total before sample analysis. Any concern of sample degradation?

Response: Great point. The baseline serology testing was completed by March of 2017 so we were not concerned about sample degradation. We added the following clarification to the methods: "The samples were stored at -20°C until all sample collection for the entire study visit was completed and then shipped at ambient temperature to the Centers for Disease Control and Prevention (CDC) in Atlanta, GA, where they were stored at -20°C until testing between February and March of 2017, approximately 12 months after collection."

Distance to water. When community leaders listed all sources of water, did they also describe type of source, name? Was there much heterogeneity in the number of sources per village, any effort made to validate community leader report to actual on-the-ground use of water sources by households? Please clarify, given potential issues of recall reliability and thoroughness. How did the census workers know they had reached the water source described by the community leaders? Were they accompanied to the source?

Response: When the community leaders list the water points, the team members ask about water point type and name. The team members also go to each water point during each census visit. They take GPS, select the water point type, and take a picture of the water point. They are very often accompanied by a community guide. If a guide is not present, they will ask nearby households to help guide them to the correct water point. There was an average of 2.7 water points per community (SD 1.6). This information is provided in the "Distance to water" section. We added the following sentence to this section: "Census workers were accompanied by the community leader or a community representative."

GPS point -- was a minimum accuracy achieved before recording the waypoint? (e.g. <10m?)

Response: We did not record GPS accuracy for household are water point GPS coordinates during the study visit. We did record accuracy during later study visits for the trial. At the 24-month study visit the mean accuracy was 16.7 meters (SD 22.9). We added the following sentence to the methods: "Census workers recorded the name, sex, and age of each household member and the GPS coordinates of the house (accuracy of GPS approximately 15-20m)."

"and thus used distance to the nearest water source (improved or unimproved) for the analysis." So basically making the assumption that this is a proxy for water quantity? This should be included as a limitation.

Response: We agree that this is a limitation of the study. The following sentences describe this limitation in the discussion: "Finally, we opted to measure distance to the nearest protected or unprotected water source to evaluate the effect of water quantity on enteropathogen and C. trachomatis transmission. To evaluate the effect of water quality on enteropathogen transmission, distance to the

nearest protected water source may have been a more appropriate exposure."

Statistical analysis -
"For the remaining antigens we used finite mixture models to fit Gaussian distributions for the log10 transformed MFI-bg values [10,22] and determined the seropositivity cutoffs using the mean plus three standard deviations of the first component." Does the Priest et al. paper use finite mixture models, ? Please clarify. If no, is there any paper you can cite to justify this approach.

Response: Apologies for the confusion, we had an error with our reference software and all the references were mismatched. We have corrected the reference numbering in the revision. The following references apply to this method:

****Arnold BF, Martin DL, Juma J, Mkocha H, Ochieng JB, Cooley GM, et al. Enteropathogen antibody dynamics and force of infection among children in low-resource settings. eLife. 2019;8: e45594. doi:10.7554/eLife.45594**

****Benaglia T, Chauveau D, Hunter DR, Young DS. mixtools: An R Package for Analyzing Finite Mixture Models. J Stat Softw. 2009;32: 1–29.**

"consistent with other enteropathogen serology in cohorts from low-resource settings" The authors cite the Priest et al. 2006 paper, but imply this finding is found in multiple cohorts. Please cite at least an additional paper demonstrating this consistency, particularly one that covers additional pathogens besides the Cryptosporidium species reported in the Priest et al. 2006 paper.

Response: we had an error with our reference software and all the references were mismatched. We have corrected the reference numbering in the revision.The following references apply to this statement:

****Arnold BF, Martin DL, Juma J, Mkocha H, Ochieng JB, Cooley GM, et al. Enteropathogen antibody dynamics and force of infection among children in low-resource settings. eLife. 2019;8: e45594. doi:10.7554/eLife.45594**

"For pathogens with presumed lower transmission based on more slowly rising age-dependent seroprevalence (C. trachomatis and S. enterica)"
Is there a paper that can be cited to justify this presumption?

Response: We added the following citation to support this statement:

****Arnold BF, van der Laan MJ, Hubbard AE, Steel C, Kubofcik J, Hamlin KL, et al. Measuring changes in transmission of neglected tropical diseases, malaria, and enteric pathogens from quantitative antibody levels. PLoS Negl Trop Dis. 2017;11: e0005616. doi:10.1371/journal.pntd.0005616**

"Among the 33% of children whose household was randomly selected for inclusion in the household survey, we adjusted for socio-economic status (SES) using an indicator variable calculated using a principal component analysis." Meaning this is a sensitivity test? Please provide more details on the variables used to construct the PCA, perhaps as supplemental material. Did you calculate quintiles?

Response: Thank you for noticing this omission. We added the following to clarify the method: "Among the 33% of children whose household was randomly selected for inclusion in the household survey, we adjusted for socio-economic status (SES) using quintiles of an asset index score calculated using a principal component analysis of the following variables: if the household had electricity, the animals owned and species, education of the head of household and if someone in the household owned a radio."

Reviewer #2: The objectives and methods of this study were very well thought out and clearly articulated. The methods were appropriate to fulfill the two primary objectives. Because this is a secondary analysis of data from another study, there was not sufficient statistical power to control for socio-economic status. However, this limitation was clearly outlined.

The predictor of interest, distance from water, was calculated simply as the median linear distance from households in a community to the nearest water source. There are many limitations to this definition, and they are adequately explained in text. However, although the authors clearly stated their rationale for ignoring water quality in this analysis, I think that it would improve the paper to take water quality into account.

The analysis does not take into account whether water sources are improved or unimproved. Given that information on whether water sources are improved or unimproved is available, the authors should test whether access to improved water is independently associated with seroprevalence, and whether it is a possible confounder in the relationship between distance to water and seroprevalence (is access to improved water associated with both distance from water and seroprevalence of C trachomatis and/or GI pathogens of interest?). It is plausible that transmission of C trachomatis is associated primarily with quantity of water, but transmission of GI pathogens can be linked to poor quality water.

Response: Thank you for reviewing this paper and for your valuable insights. We agree that the quality of the water source is an important factor to consider. In this analysis we prespecified that we would look at distance to unimproved and improved water sources to investigate the effects of water quantity rather than water quality. To investigate the association with water quality we considered estimating distances to the nearest improved water source but many communities had no improved water source. In these cases, distance to the nearest improved source would be very far (to the next nearest community with an improved water source) and was almost certainly not a water source that was actually used by those households because of the far distance. For this reason, we felt that distance to the nearest water source would be the most valid analysis in the present context.

We used causal inference framework to adjust for potential confounders. We agree that the type of water source (improved/unimproved) causes the outcome (exposure to certain pathogens), however the type of water source does not cause the exposure (distance to the nearest improved/unimproved water source) and thus it would not be appropriate to include this variable as a confounder.

Reviewer #3: Methods / Study population: It would be helpful to clarify how childrens ages were recorded (years, months, days?).

Response: We added the following clarification sentence: “Age was calculated from the date of birth if known or the child’s age in years for children older than one year and in months for children one year old and under.”

Methods / Covariates: Please provide the rationale behind only assessing socioeconomic status in one-third of households. Also, please provide detail or references to how the household status questionnaires were designed and how the factors presented in the results (such as having a mobile phone or a radio) compare to socioeconomic status and human behaviour.

Response: Additional details about the household survey are available in the methods paper describing the parent trial [Fry DM, Aragie S, Melo JS, Aiemjoy K, Chanyalew M, Freeman M, et al. WASH Upgrades for Health in Amhara (WUHA): design and rational for a cluster-randomized trial in Ethiopia. Submitted 04/2020. BMJ Open]. The random third of households rather than all households were interviewed because of budget limitations and are described in the methods paper for the parent trial.We added a citation for this paper and the following sentence to the methods “The survey was limited to a subset of households for budgetary reasons; additional details on the household survey are available elsewhere [16].”

Methods / Laboratory methods: Please add a sentence describing why these particular enteropathogens were selected for study.

Response: We added the following sentence to the methods: “The enteropathogens were selected on the basis of antigen availability and known circulation in the region.”

Methods / Laboratory methods: Please provide more information about the “two positive controls”. Are these serum samples with known exposure to all pathogens in the panel, or a composite reference of multiple sera? How were they defined as positive? A citation would suffice if this has been published elsewhere.

Response: We pooled together 4 different serum samples to make the positive control dilutions. Antibody status for each antigen for each serum sample was determined by previous testing on multiplex. However, infection status was not always known. Therefore, the controls served as process indicators rather than markers of test validity. The “high positive” control was a 1:100 dilution of the serum pool and the “low positive” control was a 1:1000 dilution of the serum pool. This is so we could have various ranges of MFI values for each antigen to monitor over time to make sure we had consistency between plates. We added the following language to clarify the methods: *“Multiplex bead assay:* Each 96-well plate included a buffer-only blank, one negative control, and two positive controls. The two positive control wells contained pooled serum that was previously classified as seropositive for each antigen at two dilutions: 1:100 and 1:1000.”

Methods / Statistical analysis / Calculation of age-dependent seroprevalence section: For the benefit of the non-statistician audience of this paper, suggest adding a few sentences describing the analysis approach in lay terms and why it is beneficial over more simplistic approaches.

Response: Thank you for this suggestion. We added the following sentences to the methods which we hope will add clarity for the readers: "The relationship between age and seroprevalence is usually non-linear and varies by infection dynamics. Therefore, we sought a flexible modeling approach that does not impose assumptions on the functional form of the relationship between age and seropositivity."

Results

-Does the analysis presented match the analysis plan?

-Are the results clearly and completely presented?

-Are the figures (Tables, Images) of sufficient quality for clarity?

Reviewer #1: "51.3% (1169/2267) of children were female"
Please double-check this – I get 51.6% when using 1169/2267 (as reported in Table 1)

Response: Thank you for catching this typo. We corrected the percentage to 51.6%

Table 1 – I suggest reporting meters to nearest whole meter given GPS accuracy issues.

Response: Thank you for this suggestion, we updated the rounding of meters to the nearest whole meter.

Surface water types: preferable to report according to JMP classifications if possible, e.g. surface water instead of open water, unprotected dug well instead of unprotected well.

Response: Thank you, we updated the classifications accordingly.

Table 1 – animal ownership; possible to separate out by cows, chickens, etc? Or those kept in compound?

Response: Unfortunately, the data were not tagged by discrete animal type.

Possible to include anything related to handwashing or sanitation facilities? Occupation? Education level?

Response: For the majority of households (85.2% (643/755)), the primary occupation was agricultural work. We added this information to the table.

Table 1 – for subset with household survey, are these nearest water sources still based on the list generated by the community leaders? Or are these water sources according to household survey answers? If the latter, did these source types match up with the

source type indicated according to the method you used for determining nearest water source?

Response: These are still based on the list generated by the community leaders to be consistent. We added the following sentence to the methods to clarify: "Distance to the nearest water source was calculated in the same way as above for the subset of households with the household survey."

In the household survey, did you collect round-trip travel time to go to source, collect water, and return?

Response: We did collect this information in the subset with the household survey. But as you eluded to above the water source used for the analysis was the one nearest to the household measured via GPS even for households participating in the survey to be consistent with the 2/3 of households for whom we did not have the water source or distance/time measured.

Figure 1 - I might have missed it, but what might explain the waning seroprevalence of G. intestinalis as age increases after 2 years? Does it become a long-term sub-clinical infection with a corresponding reduction in antibody response? It appears much different than the other pathogens, and I think it deserves some more discussion.

Response: The underlying immunology is likely pathogen-specific, but Giardia has been shown to exhibit increasing infection prevalence with age (many infections asymptomatic) in other cohorts in low-resource settings (Platts-Mills et al. 2018), suggesting that the IgG response is weaker at older ages despite infection. The precise immunological mechanism for lower mean IgG levels among older ages is not currently known, but has been observed in multiple other cohorts. For example, Arnold et al. 2019 demonstrated declining mean IgG with age for Giardia (VSP-3, VSP-5), ETEC (LTB) and Campylobacter (p18, p39) in cohorts from Haiti and Kenya. Age-dependent antibody kinetics in that study suggest that much of the decline of mean IgG with age for these pathogens was likely due to acquired immunity, which results in either lower rates of infection, or more likely, if children are infected they experience less severe disease (and potentially a less robust IgG boost).

Platts-Mills, J. A., Liu, J., Rogawski, E. T., Kabir, F., et al.. Use of quantitative molecular diagnostic methods to assess the aetiology, burden, and clinical characteristics of diarrhoea in children in low-resource settings: a reanalysis of the MAL-ED cohort study. Lancet Glob Health (2018). doi:10.1016/S2214-109X(18)30349-8

Arnold, B. F., Martin, D. L., Juma, J., Mkocha, H., et al.. Enteropathogen antibody dynamics and force of infection among children in low-resource settings. *Elife* 8, (2019).

We added the following text to the discussion: "Unlike for other pathogens in the study, G. intestinalis seroprevalence declined after age two years. Giardia has been shown to exhibit increasing infection prevalence with age in other cohorts in low-resource settings with a high proportion of asymptomatic infections [48], suggesting that the IgG response is weaker at older ages despite infection. The precise immunological mechanism for lower mean IgG levels among older ages is not currently known, but the phenomena has been observed in multiple other cohorts. For example, Arnold et al. demonstrated declining mean IgG with age for Giardia (VSP-3, VSP-5), ETEC (LTB) and Campylobacter (p18, p39) in cohorts from Haiti and Kenya [13]. Age-dependent antibody kinetics in that study suggest that much of the decline of mean IgG with age for these pathogens is likely due to

acquired immunity, which results in either lower rates of infection, or more likely, if children are infected they experience less severe disease and potentially a less robust IgG boost."

Also, regarding age-dependent seroprevalence patterns for S. enterica and C. trachomatis in contrast to C. jejuni, ETEC, E. histolytica, and C. parvum -- could this variation be somehow linked to child and/or caretaker behavior, or household-specific factors, in addition to low vs. high transmission more generally? Evidence of this phenomenon in other studies?

Response: Since many of the enteric pathogens included in this study likely share common transmission pathways, and would be similarly influenced by child-, caretaker-, and household-specific characteristics, the slower rise of age-dependent seroprevalence likely reflects lower transmission overall in the community. There are no specific characteristics that we could think of in the present context that would have differentially protected children in this age group from *S. enterica* and *C. trachomatis* that would have not also influenced transmission of other pathogens.

Also, the benefits of delayed seroconversion for an individual (e.g. growth, stronger immune system once pathogen is eventually encountered, improved cognitive development?) might be worth highlighting, as any associations become a little less noteworthy to policymakers if everyone is going to seroconvert eventually. However, is there any evidence that delayed seroconversion could actually result in a stronger immune response at an older age resulting in more severe illness?

Response: For pathogens to which children are potentially exposed from birth, a shift of the age-dependent seroprevalence curve toward one that rises more slowly and plateaus at a lower level reflects lower overall transmission (Arnold et al. 2017, Arnold et al. 2019). For example, comparisons of age-dependent IgG from many enteric pathogens among children living in Haiti and the United States show that for most pathogens mean IgG levels (and related seroprevalence) in the United States never reach the same level as they do in Haiti. Since enteric pathogen IgG responses following infection are almost certainly not elevated for life (IgG response remains elevated for permanently immunizing pathogens such as measles), populations with low transmission would not be expected to approach 100% seroprevalence. Experience of fewer enteric infections early in life (including subclinical infections, as would be detected through IgG response) has been shown to be associated with slightly improved linear growth during the first two years of life (Rogawski et al. 2018). To our knowledge, it is unknown what the longer-term impact of such infections might be on cognitive development.

Arnold, B. F., Martin, D. L., Juma, J., Mkocha, H., Ochieng, J. B., Cooley, G. M., Omore, R., Goodhew, E. B., Morris, J. F., Costantini, V., Vinjé, J., Lammie, P. J. & Priest, J. W. Enteropathogen antibody dynamics and force of infection among children in low-resource settings. Elife 8, (2019).

Arnold, B. F., van der Laan, M. J., Hubbard, A. E., Steel, C., Kubofcik, J., Hamlin, K. L., Moss, D. M., Nutman, T. B., Priest, J. W. & Lammie, P. J. Measuring changes in transmission of neglected tropical diseases, malaria, and enteric pathogens from quantitative antibody levels. PLoS Negl. Trop. Dis. 11, e0005616 (2017).

Rogawski, E. T., Liu, J., Platts-Mills, J. A., Kabir, F., Lertsethtakarn, P., Siguas, M., Khan, et al. Use of quantitative molecular diagnostic methods to investigate the effect of enteropathogen infections on linear growth in children in low-resource settings: longitudinal analysis of results from the MAL-ED cohort study. Lancet Glob Health (2018). doi:10.1016/S2214-109X(18)30351-6

Figure 1 caption: “For pathogens with more than one antigen, positivity to antigen was considered positive.”
Revise to state “…positivity to EITHER antigen was considered positive”, correct? Same for Figure 3 caption, and Supplemental Figure 2 caption.

Response: Yes, thank you for catching that. We corrected the text to include “either”

“There was no indication for trend in community-level seroprevalence by community-level median distance to the nearest water source”
How much variability was there within a community to nearest water source? You’ve presented the medians, but is there anything else you could show, and might that be predictive of village seroprevalence heterogeneity among children <3?

Response: Thank you for this interesting suggestion. We calculated the inter quartile range of distance to the nearest water source for each community and then summarized the median of this which was 229 meters. We then investigated if there was an association between the community IQR in distance to the nearest water source and the prevalence for each pathogen. There was no apparent association. We have made no changes to the manuscript.

Was there any association between water source type and distance to water source? SES and distance? Was there any association between animal ownership and distance? Would that be something to adjust for? Was animal ownership part of the SES index? Could those who live futher from water sources have more animal exposures?

Response: Animal ownership was part of the SES index. We clarified the PCA components with the following addition:“Among the 33% of children whose household was randomly selected for inclusion in the household survey, we adjusted for socio-economic status (SES) using quintiles of an indicator score calculated using a principal component analysis of the following variables: if the household had electricity, the animals owned and species, education of the head of household and if someone in the household owned a radio.”

With the SES variables measured in just 33% of household we lacked the power to delve into associations between SES and distance.

Why not adjust for crowding, sanitation and hygiene indicators if testing hypothesis of water source distance resulting in higher exposure to C. trachomatis and enteric pathogens?

Response: A very interesting suggestion, but again with only 33% of households with SES indicators and household-level associations we lacked the power to investigate these associations.

Reviewer #2: The results are very clearly presented and match the described methods. The figures were exceptionally well done and clear.

Reviewer #3: (No Response)

Conclusions

-Are the conclusions supported by the data presented?

-Are the limitations of analysis clearly described?

-Do the authors discuss how these data can be helpful to advance our understanding of the topic under study?

-Is public health relevance addressed?

Reviewer #1: Discussion, second paragraph: can you infer active infection from higher antibody count?

“To evaluate the effect of water quality on enteropathogen transmission, distance to the nearest protected water source may have been a more appropriate exposure.” I fail to see how calculating distance to the nearest protected water source vs all water sources as was done would help in evaluate effect of water quality on enteropathogen transmission, since it would be hard to make the case that households were overlooking nearer water sources of worse quality for non-drinking purposes. Wouldn’t this just increase the distance to water source only for those households that happened to have an unimproved as their nearest source? Also, improved water sources do not necessarily imply safe water provision, and at least in one recent paper, handwashing with poor quality water is still effective. Perhaps I’m missing something, but this seems like a stretch. I would say, it would be better to actually measure the water quality (ideally longitudinally).

Response: Thank you for the great insight, we agree with your suggestions and have updated the text as follows: “Finally, we opted to measure distance to the nearest protected or unprotected water source to evaluate the effect of water quantity on enteropathogen and *C. trachomatis* transmission. An alternative approach to evaluate the effect of water quality on enteropathogen transmission would be to assess the type of water source that was used by each household, measure the distance to that source and then evaluate associations between distance, water source type and seroprevalence. The ideal study would evaluate the microbiological water quality of the sources longitudinally.”

“Analyzing qualitative antibody levels is an alternative to seroprevalence that may retain the higher resolution needed in high-transmission settings [9].”
You mean quantitative, not qualitative, correct? Please revise.

Response: Thank you, we revised.

“The study site region has tremendous gradation in altitude, with many high plateaus and steep valleys.” There are methods for doing ansiotropic models for travel time that utilize land types and elevation changes, usually in context of health facility accessibility, but could be applied to water source accessibility, e.g. Access MOD

https://www.accessmod.org/ It would be worth mentioning future studies could use more sophisticated approaches to overcome the limitations of using Euclidean distance as a proxy for water source access. On the other hand, it might be better than reported travel time, and can be a decent proxy for route distance (Ho et al. 2014 https://doi.org/10.2166/wh.2013.042)

Response: Thanks for this suggestion. We added the following sentence to the discussion: "Future studies may consider alternative methods for calculating distance that accommodate land type and elevation changes."

Additionally, it might be worth mentioning alternative approaches to measuring water quantity, since that seems central to your hypothesis; e.g. could use sensors or measure reported number of jerricans fetched by size, per day or per week. Also worth noting that water quantity and usage for hygiene, along with water source availability as you've already pointed out, likely varies by season. Others have made the argument that focusing on household water quantity and amount used for hygiene purposes may be more predictive of trachoma and other disease risk. (Stelmach and Clasen 2015, Altherr 2019)

Response: Thank you for this suggestion. We added the following to the limitations section: "Third, we assumed that distance to the nearest water source was associated with the quantity of water used by the household. Future studies could use sensors or measure the reported number of jerrycans used over time to more precisely measure water quantity."

Supplemental Figure 1 caption – correct typos (recever and Entomoeba)

Response: Thanks - fixed

Supplemental Figure 2 caption – perhaps I missed it, but I was a little surprised to see "2328 blood samples from 2328 children aged 0 to 9 years" when everywhere else it was "2267 blood samples from 2267 children" Please clarify.

Response: This was a typo, thanks for catching it. The correct number is 2267

Reviewer #2: The conclusion section is well-written and includes a clear description of limitations.

Reviewer #3: Discussion: "The seroprevalence of trachoma" typo, please change to sero prevalence of C. trachomatis.

Response: Thank you for catching this. We corrected the text as suggested.

Discussion: Can authors comment on the decrease in age specific seroprevalence on Giardia between the ages of 2 and 9 years? And the decrease in ETEC seroprevalence between 5 and 9 years? Is this seroreversion?

Response: Yes, the decline in seroprevalence likely reflects seroreversion for these pathogens. Please see our response above to Reviewer 1 (above) for a more detailed response and explanation to this comment. Our response is also repeated here:

Response: The underlying immunology is likely pathogen-specific, but Giardia has been shown to exhibit increasing prevalence with age (many infections asymptomatic) in other cohorts in low-resource settings (Platts-Mills et al. 2018), suggesting that the IgG response is weaker at older ages despite infection. The precise immunological mechanism for lower mean IgG levels among older ages is not currently known, but has been observed in multiple other cohorts. For example, Arnold et al. 2019 demonstrated declining mean IgG with age for Giardia (VSP-3, VSP-5), ETEC (LTB) and Campylobacter (p18, p39) in cohorts from Haiti and Kenya. Age-dependent antibody kinetics in that study suggest that much of the decline of mean IgG with age for these pathogens is likely due to acquired immunity, which results in either lower rates of infection, or more likely, if children are infected they experience less severe disease (and potentially a less robust IgG boost).

Platts-Mills, J. A., Liu, J., Rogawski, E. T., Kabir, F., Lertsethtakarn, P. et al. Use of quantitative molecular diagnostic methods to assess the aetiology, burden, and clinical characteristics of diarrhoea in children in low-resource settings: a reanalysis of the MAL-ED cohort study. Lancet Glob Health (2018). doi:10.1016/S2214-109X(18)30349-8

Arnold, B. F., Martin, D. L., Juma, J., Mkocha, H., Ochieng, J. B., Cooley, G. M., Omore, R., Goodhew, E. B., Morris, J. F., Costantini, V., Vinjé, J., Lammie, P. J. & Priest, J. W. Enteropathogen antibody dynamics and force of infection among children in low-resource settings. *Elife* 8, (2019).

Editorial and Data Presentation Modifications?

Use this section for editorial suggestions as well as relatively minor modifications of existing data that would enhance clarity. If the only modifications needed are minor and/or editorial, you may wish to recommend “Minor Revision” or “Accept”.

Reviewer #1: Minor comments are as follows, perhaps a little challenging to follow without line numbering, but hopefully doable:

Last sentence of author summary: something missing here. “the relationship [OF?] water, sanitation and hygiene related exposures [TO?] disease transmission”

Response: Thank you for catching this. We fixed the wording as follows: Integrated serosurveillance is a promising avenue to explore the complexities of multi-pathogen exposure as well as to investigate associations between water, sanitation, and hygiene related exposures and disease transmission.

Background, paragraph 1, first sentence: do either reference 1 or 2 describe burden of diarrhea? Consider citing GBD or other review as background for diarrhea. Also, are diarrhea and trachoma causes of morbidity, or the actual morbidity? Wouldn’t causes be the upstream determinants resulting in these two conditions? Consider rephrasing.

Response: We had a glitch with our reference software. The references should be:

1. Flaxman SR, Bourne RRA, Resnikoff S, Ackland P, Braithwaite T, Cicinelli MV, et al. Global causes of blindness and distance vision impairment 1990-2020: a systematic review and meta-analysis. Lancet Glob Health. 2017;5: e1221–e1234. doi:10.1016/S2214-109X(17)30393-5

2. Troeger C, Blacker BF, Khalil IA, Rao PC, Cao S, Zimsen SR, et al. Estimates of the global, regional, and national morbidity, mortality, and aetiologies of diarrhoea in 195 countries: a systematic analysis for the Global Burden of Disease Study 2016. The Lancet Infectious Diseases. 2018;18: 1211–1228. doi:10.1016/S1473-3099(18)30362-1

Also, rephrased the first sentence as follows: Diarrhea and trachoma typically afflict the world's poorest populations and are major contributors to preventable morbidity [1,2].

Background, paragraph 1, last sentence: Is this the correct reference for the statement? Please consider citing additional papers that address water quantity, use for hygiene, and distance from water source.
Eg. https://doi.org/10.1016/j.wrr.2014.04.001

Also specific to Ethiopia: Gibson and Mace 2006
https://journals.plos.org/plosmedicine/article?id=10.1371/journal.pmed.0030087

Also, water fetching time and trachoma risk: Altherr 2019
https://link.springer.com/article/10.1186/s13071-019-3790-3

Consider also this review by Cassivi 2019 https://doi.org/10.1016/j.ijheh.2019.06.011 and/or Overbo 2016: https://doi.org/10.1016/j.ijheh.2016.04.008 … note distinction between water-washed and waterborne infections, which you may want to mention with respect to the panel you've examined.

Response: We had a glitch with our referencing software. The correct reference is:

****Stelmach RD, Clasen T. Household Water Quantity and Health: A Systematic Review. International Journal of Environmental Research and Public Health. 2015;12: 5954–5974. doi:10.3390/ijerph120605954**

We also added the suggested references.

"a longer window to identify exposed individuals" Just curious -- does exposure to the pathogens under study necessarily result in seroconversion, development of antibodies, do they wave over time? Or might the dose influence likelihood of infection and seroconversion? Is there any literature that you can cite on this, perhaps in the discussion, could this be a limitation? Or maybe "infected individuals" could be more accurate than "exposed individuals"?

Response: Development of an IgG response to the pathogens reflects an infection, but the IgG levels wane over time since infection. The precise post-infection IgG kinetics are currently unknown for the pathogens included in this study, but best-estimates to date for IgG half-life for the enteric pathogens included in the study is between 51 and 169 days (Priest et al. 2001, Arnold et al. 2019); for *C. trachomatis*, the IgG half-life is likely considerably longer (Goodhew et al. 2014), and over this age range IgG response likely reflects a cumulative incidence of infection. We chose to use the "exposed individuals" nomenclature throughout the manuscript because elevated IgG response does not necessarily reflect active infection — children could have a current infection or have had a previous infection. Estimating time since infection requires understanding how

IgG wanes with time since infection, which is currently an open area of research for all the pathogens in this study.

**** Goodhew, E. B., Morgan, S. M. G., Switzer, A. J., Munoz, B., Dize, L., Gaydos, C., Mkocha, H., West, S. K., Wiegand, R. E., Lammie, P. J. & Martin, D. L. Longitudinal analysis of antibody responses to trachoma antigens before and after mass drug administration. *BMC Infect. Dis.* 14, 216 (2014).**

Background , second paragraph, 4th sentence. Antibody response generally? Or quantitative antibody response more specifically, i.e. has the potential to differentiate current (regardless of symptomology?) vs past infection? A little confusing what is meant by "enumerates"… I'm not sure if you've provided sufficient evidence to back up this claim, and I don't believe the Lammie 2012 paper discuses symptomatic vs asymptomatic infections and corresponding antibody response. Please clarify and/or rephrase.

Response: Apologies, this was not the correct reference. Here is the correct one:

Arnold BF, van der Laan MJ, Hubbard AE, Steel C, Kubofcik J, Hamlin KL, et al. Measuring changes in transmission of neglected tropical diseases, malaria, and enteric pathogens from quantitative antibody levels. PLoS Negl Trop Dis. 2017;11: e0005616. doi:10.1371/journal.pntd.0005616

Reviewer #2: 1. It was not mentioned in the methods section that the community-level distance to water was defined as median distance to water of surveyed households in the community. This should be added.

Response: We added the following sentence to the methods: We calculated community-level distance to water as the median distance from each household in the community to its nearest water source.

2. The sentence "There was indication for correlation between C. trachomatis and E. histolytica, ETEC, C. jejuni and S. enterica (Pearson correlation > 0.3)" (first full sentence p 12) is difficult to understand. The authors should clarify whether this sentence is referring to the pairs of pathogens listed as correlated in Supplemental Figure 2 and, if so, to which pairs of pathogens it is referring.

Response: We agree this sentence was confusing. We clarified as follows: "There was indication for pair-wise correlation in community level seroprevalence between *C. trachomatis* and *E. histolytica*, ETEC and *S. enterica*, *C. jejuni* and C. parvum, and C. parvum and E. histolytica (Pearson correlation > 0.3) (Supplemental Figure 2)."

3. It is mentioned in text that seroprevalences of ETEC and C trachomatis were higher in the fourth quartile compared to the first quartile but the results are not statistically significant (second sentence, last paragraph, p 12). Although this is a true assertion, it is also true that the seroprevalences of E histolytica and C parvum were higher in the first quartile compared to the fourth quartile, but this was also not statistically significant. The authors should either remove mention of results that were not statistically significant or make equal mention of results that do and do not support their hypothesis. Alternatively, they could provide an explanation for why the ETEC and C trachomatis results were more noteworthy than the E histolitica and C parvum results.

Response: Thank you for the observation, we agree that it would be best to avoid selective reporting of non-significant results and have removed this sentence in the revision.

4. In the third sentence in the last paragraph on page 16, "qualitative" was used when I believe that they authors meant to say "quantitative."

Response: Thanks, yes you are correct.

5. In the second sentence of the first paragraph on page 17, ETEC was mentioned as a pathogen for which seroprevalence was higher in the 4th quartile versus the 1st. Given my previous comments, I think this mention should be removed.

Response: Yes, you are correct – we removed ETEC from this sentence.

Reviewer #3: Abstract / Methods: “1-9 years” typo? Methods section says 0-9.

Response: Thank you for catching this. It should be 0-9 years.

Author summary: “Trachoma, and infection of the eye” typo

Response: Corrected to “Trachoma, an infection”

Background: “Increased access to water for food preparation and washing of hands, faces, and clothing is hypothesized to reduce transmission of both infectious diarrhea and C. trachomatis [3–6].” There might be a better reference than the pinsent one for influence of water access on transmission of C. trachomatis.

Response: We had a glitch with our referencing software, the correct references are as follows:

3. Emerson PM, Cairncross S, Bailey RL, Mabey DC. Review of the evidence base for the “F” and “E” components of the SAFE strategy for trachoma control. Trop Med Int Health. 2000;5: 515–527. doi:10.1046/j.1365-3156.2000.00603.x

4. Taylor HR, Burton MJ, Haddad D, West S, Wright H. Trachoma. Lancet. 2014;384: 2142–2152. doi:10.1016/S0140-6736(13)62182-0

5. Fewtrell L, Kaufmann RB, Kay D, Enanoria W, Haller L, Colford JM. Water, sanitation, and hygiene interventions to reduce diarrhoea in less developed countries: a systematic review and meta-analysis. The Lancet Infectious Diseases. 2005;5: 42–52. doi:10.1016/S1473-3099(04)01253-8

6. Waddington H, Snilstveit B. Effectiveness and sustainability of water, sanitation, and hygiene interventions in combating diarrhoea. Journal of Development Effectiveness. 2009;1: 295–335. doi:10.1080/19439340903141175

Summary and General Comments

Use this section to provide overall comments, discuss strengths/weaknesses of the study, novelty, significance, general execution and scholarship. You may also include additional comments for the author, including concerns about dual publication, research ethics, or publication ethics. If requesting major revision, please articulate the new experiments that are needed.

Reviewer #1: Thank for the opportunity to review this paper. It was very well written and I found it an interesting and enjoyable read.

My comments are fairly minor, although in general, I think the authors need to provide

more justification for the hypothesis and the biologic plausibility, paying particular regard to each of the pathogens under study and how they may differ according to transmission patterns, risk factors, and relation to water quantity. With a relatively crude indicator of water quantity (nearest source) assumed to correspond to water source distance, further assumed to be the nearest source used by that particular house according to a community leader's tally, the potential role of unmeasured confounders should at least be noted. Potentially relevant determinants of both risk of exposure and susceptibility to infections could include water source reliability, water fetching and animal tending responsibilities, soil contact/ingestion, child feces management, household and community-level hygiene and sanitation practices, water treatment and storage practices, nutrition status, and other comorbidities. In future work, it could also be helpful to look for spatial clustering of the diseases of interest, particularly among children less than 3 years of age before seroprevalence (typically) plateaus, though I believe that is likely beyond the scope of this paper. I also think that the multiplicity of benefits (time-savings, improved safety, reduced musculoskeletal injury, stress, etc.) that comes with safe and more consistent and proximal water sources should be highlighted in the closing remarks of the discussion, as potential reductions of pathogen risk shouldn't be considered in isolation. In short, this is an important piece of work and I applaud the authors' efforts.

Response: Thank you for your valuable insights and comments. We really appreciate the time you took to review this manuscript and the attention to detail. We agree with your assessment that distance to the nearest water source is a crude measure of water quality and highlighted this as a limitation in the discussion. We also added the following sentences describing the potential for unmeasured confounding to the discussion:

"The association between water quality and risk of exposure and susceptibility to infections is subject to many potential confounding variables that we were unable to measure such as household and community level hygiene and sanitation practices and water treatment and storage practices. Future studies should consider measuring and evaluating these variables."

Reviewer #2: I appreciate the opportunity to review this manuscript. It describes an important study with results that have the potential to be used for advocacy for better water access in Ethiopia and elsewhere. The research was well-presented with clear and well-constructed tables and graphs.

Other than the minor editorial concerns listed above, my only suggestion for the authors is to investigate whether water quality was a confounder to the relationship between distance to water and transmission of C trachomatis and/or GI pathogens. It appears that this analysis can be conducted with available data.

Response: Thank you for your valuable comments, we addressed your suggestion to investigate water quality in the comments above [Methods, reviewer #, response 1].

Reviewer #3: The authors present the data from serological analysis of ~2200 children in Ethiopia and assess the correlation between seropositivity and proximity to water source. In a smaller subset, they investigate more household variables related to socioeconomic status. The study is conducted in an area of high infectious disease burden, an appropriate context to utilise this type of tool, and the results are presented

well. There are weaknesses to the manuscript, but these are for the most part acknowledged in the discussion. I believe the manuscript should be accepted for publication, pending the consideration of some minor points below.

Response: Thank you for your time reviewing this manuscript and for your valuable comments.
